# The effect of thermocyclic aging on color stability of high translucency monolithic lithium disilicate and zirconia ceramics luted with different resin cements: an in vitro study

**DOI:** 10.1186/s12903-021-01963-9

**Published:** 2021-11-19

**Authors:** Linah M. Ashy, Adnan Al-Mutairi, Tariq Al-Otaibi, Lulwa Al-Turki

**Affiliations:** 1 Department of Oral and Maxillofacial Prosthodontics (OMP), King Abdulaziz University Faculty of Dentistry (KAUFD), P.O. Box 80209, Jeddah, 21589 Saudi Arabia; 2 Ministry of Health, Jeddah, Saudi Arabia; 3 University Dental Hospital, Jeddah, Saudi Arabia

**Keywords:** High-translucency ceramics, Color stability, Zirconia, Lithium disilicate, Resin cements

## Abstract

**Background:**

High-translucency monolithic zirconia were developed to combine the esthetics of all ceramic restorations with the strength properties of zirconia. The purpose of this study was to compare the color stability of high-translucency monolithic zirconia ceramics with lithium disilicate luted using light-cure versus dual-cure resin cements following thermocyclic aging.

**Methods:**

Forty specimens, each composed of 10 × 10 × 1 mm ceramic slice luted to dentin surface of an extracted tooth, were prepared and assigned into four groups (*n* = 10) as follows; LiDi/LC: lithium disilicate luted by light-cure resin cement; LiDi/DC: lithium disilicate luted by dual-cure resin cement; Zr/LC: zirconia luted by light-cure resin cement; and Zr/DC: zirconia luted by dual-cure resin cement. Color analysis of the specimens was performed before and after 3000 thermal cycles by means of spectrophotometry. The CIE L*a*b* values of the specimens were measured, and data were analyzed statistically at a significance value of *p* < 0.05.

**Results:**

Thermocycling resulted in a significant change in color coordinates of specimens with an overall *ΔE* = 3.59 ± 1.60, but there was no statistically significant difference in the color change value among all tested groups (*P* = *0.756*).

**Conclusions:**

At 1 mm restoration thickness, the color stability of high-translucency monolithic lithium disilicate and zirconia ceramics were not significantly different irrespective of the cement type used.

*Clinical implication* Understanding the difference in color stability of dental ceramics may help in determining long-term esthetic result.

## Background

As the expected standards of the public in the area of dentistry have increased, so has the need for tooth-colored restorations with superior properties. Consequently, various tooth-colored restorations with superior esthetic and mechanical qualities have been developed. Lithium disilicate glass ceramics are commonly used nowadays due to their high strength, adequate bonding to tooth structure, easy construction procedure, and acceptable esthetic appearance [1]. Nevertheless, dental material manufacturers have developed high-translucency monolithic zirconium oxide restorations to combine the esthetics of all ceramic restorations with the superior strength properties of zirconia [2].

The optical and mechanical properties of highly translucent ceramic materials have been compared in few recent studies [2, 3]. Church et al. [2] reported in an in vitro study that a lithium disilicate material (IPS e.max CAD) has greater translucency than four other types of high-translucency monolithic zirconia. These were BruxZir shaded 16, InCoris TZIC, Lava Plus, and BruxZir HT, all being of the same thickness. Similarly, Yan et al. [3] found that the translucency of lithium disilicate is superior to those of both, 4-Y-PSZ (4 mol% yttria partially-stabilized zirconia) and 3-Y-PSZ, whereas it was insignificantly different from that of 5-Y-PSZ.

Color stabilities of monolithic ceramics and composite resin cements have also been reported. Putra et al. [4] have reported a minimal change in the percentage of light transmittance of BruxiZuir anterior solid zirconia, super-translucent Katana zirconia and ultra-translucent Katana zirconia, after a period of hydrothermal aging at 134 °C and 0.2 MPa. However, a change in color after artificial accelerated aging was reported for resin nano-ceramics, feldspathic ceramics, and leucite ceramics cemented with dual-cure resin cement to extracted teeth [5]. Moreover, the change in the color of the ceramic material was found to be directly related to the alteration in the color of the associated luting resin cement, where the thinner the restoration (0.5 mm), the greater was the reported color change when dual-cure or light-cure cements were used [6]. Mina et al. [7] studied the color stability after the accelerated aging of four different types of luting resin cements and reported a significant color change in Nexus 3 dual-cure cement, Rely-x ultimate dual-cure cement, and Nexus 3 light-cure cement, but not for Variolink esthetic light-cure cement. To our knowledge, the color stability of high-translucency ceramics has been insufficiently investigated in the literature. Therefore, the aim of this study was to determine and compare the color stability of high-translucency monolithic lithium disilicate and zirconia ceramics when luted with light-cure and dual-cure resin cements and subjected to thermocyclic aging. The specific objectives are:

1. To determine the effect of thermocycling on the color coordinates of the luted ceramic material.
2. To determine the effect of the ceramic type and/or cement type on the color coordinates of the luted ceramic material.
3. To compare the color stability of luted lithium disilicate ceramic to that of luted zirconia ceramic.

The null hypothesis of the study was that the color stability of high-translucency monolithic lithium disilicate and high-translucency zirconia ceramics luted with light-cure or dual-cure resin cements are the same after thermocycling.

## Material and methods

### Specimens preparation

Forty extracted sound human premolars were chosen for this study after obtaining an ethical approval (#116-10-18). Teeth were extracted for orthodontics reasons. Immediately after extraction, remnants of soft tissue were removed using hand scaler. All teeth were assessed for visible fractures or discoloration. The dimensions of the occlusal surfaces of the crowns were measured using periodontal probe. Only sound teeth with 7–8 mm mesio-distal dimension and 8–9 mm bucco-lingual dimension were included in this study. Teeth roots were mounted in cylindrical mounts of self-cure acrylic resin (GC AMERICA INC). Subsequently, the teeth were stored in normal saline at room temperature. Occlusal enamel of all teeth was removed by occlusal reduction up to dentin using a diamond wheel on a high-speed handpiece (Diamond Metal Blade, ALLIED HIGH TECH PRODUCTS, INC). Forty (10 × 10 × 1 mm) ceramic slices were prepared from high-translucency monolithic lithium disilicate blocks (A1 shade) and high-translucency monolithic zirconia disc (A1 shade); twenty lithium disilicate slices (IPS e.max CAD HT, Ivoclar Vivadent) and twenty zirconia slices (Katana Zirconia STML, Kuraray Noritake Dental Inc). Ceramic blocks and disc were cut into slices using a diamond wheel of 5″ × 0.15″ × 0.5 (Diamond Metal Blade, ALLED HIGH TECH PRODUCTS, INC). Each slice was polished using silicon carbide grinding paper (macro-cut 180/p180 grit followed by micro-cut 1200/p2500 grit), then zirconia was sintered at 1550 °C for 2 h and Lithium disilicate was crystallized by firing at 770 °C for 5 min followed by firing at 850 °C for 10 min according to the manufacturer’s recommendations. The dimensions of the resultant slice were verified with a digital caliper.

The ceramic slices were luted to the dentin surface of extracted teeth using either light-cure resin cement (NX3 Nexus third generation light-cure, clear shade, Kerr) or dual-cure resin cement (NX3 Nexus third generation dual-cure, clear shade, Kerr). The materials used in this study and luting technique are described in Tables 1 and 2 respectively. A total of forty specimens were prepared and randomly assigned into four groups (*n* = 10 per group) as follows: group 1 (LiDi/LC): lithium disilicate luted by light-cure resin cement; group 2 (LiDi/DC): lithium disilicate luted by dual-cure resin cement; group 3 (Zr/LC): zirconia luted by light-cure resin cement; and group 4 (Zr/DC): zirconia luted by dual-cure resin cement. Specimens were stored in normal saline in a covered container at room temperature for 24 h before color measurement.

**Table 1:** Materials used

Material	Manufacturer	Composition
Lithium disilicate (IPS e.max CAD)	Ivoclar Vivadent	80% SiO_2_, 19% Li_2_O, 13% K_2_O, 11% 8% P_2_O_5_, 8% ZrO_2_, 5% ZnO, 5% Al_2_O_3_, 8% MgO, Coloring oxides
Zirconia (Katana Zirconia STML)	Kuraray Noritake Dental Inc	ZrO_2_ + HfO_2_ 88–93%, yttrium oxide (Y_2_O_3_) 7–10%, other oxides 0–1%
Porcelain etchant	Ultradent Products, Inc	9% buffered hydrofluoric acid
Silane	Ultradent Products, Inc	Methacryloxy propyl trimethoxy silane < 10%, Isopropyl alcohol < 95%
Gel etchant	Kerr Corporation	Phosphoric acid 35–40%, cobalt alumina blue spinel < 1%
OptiBond Solo Plus	Kerr Corporation	Ethanol 10–3%, 2-hydroxyethyl methacrylate 10–30%, 2-hydroxy-1,3-propanediyl bismethacrylate 1–5%, alkali fluorosilicates (Na) 0.1–1%
NX3 Nexus light-cure resin cement	Kerr Corporation	Glass, oxide, chemicals 30–60%, ytterbium trifluoride 10–30%, poly(oxy-1,2-ethanediyl), α,α′-[(1-methylethylidene)di-4,1-phenylene]bis [w-[(2-methyl-1-oxo-2-propenyl)oxy]-5—10%, 7,7,9(or 7,9,9)-trimethyl-4,13-dioxo-3,14-dioxa-5,12-diazahexadecane-1,16-diyl bismethacrylate 5—10%, 2,2′-ethylenedioxydiethyl dimethacrylate 5–10%, 2-hydroxyethyl methacrylate 1–5%
NX3 Nexus dual-cure resin cement	Kerr Corporation	Base: Barium aluminoborosilicate glass 30–60%, ytterbium fluoride 10 < 30%, ethoxylated bisphenol-A dimethacrylate < 15%, urethane dimethacrylate < 10%, triethylene glycol dimethacrylate < 10%, hydroxyethylmethacrylate < 10%, fumed silica < 5%, bisphenol-A diglycidyl methacrylate < 5%, ethyldimethylaminobenzoate < 0.5% Catalyst: Barium aluminoborosilicate glass 30–60%, ytterbium fluoride 10 < 30%, triethylene glycol dimethacrylate < 10%, ethoxylated bisphenol-A dimethacrylate < 10%, urethane dimethacrylate < 10%, fumed silica < 5%, bisphenol-A diglycidyl methacrylate < 5%, hydroxyethylmethacrylate < 5%, peppermint oil < 0.5%

**Table 2:** Cementation procedure

Study group	Ceramic surface treatment	Dentin surface treatment	Cementation procedure
IPS e.max CAD (groups LiDi/LC and LiDi/DC)	Etch with 9% hydrofluoric acid for 10 s	Etch with 37.5% phosphoric acid for 10 s	Apply a 2 mm diameter drop of NX3 Nexus cement onto the center of dentin surface
	Rinse thoroughly with water	Rinse thoroughly with water	Place the ceramic slice on top of the cement
	Air dry	Gentle air flush for 10 s	Apply finger pressure until excess cement appears around the tooth margins
	Apply silane for 60 s	Apply OptiBond Solo for 15 s	Light cure^a^ for 2 s
	Air dry	Air thin for 3 s	Remove the excess by explorer then light cure^a^ 20 s on each surface of the assembly
Katana Zirconia (groups Zr/LC and Zr/DC)	Sandblast with 50 μm aluminum oxide powder at 2 bar/30 psi	Light cure^a^ for 10 s	
	Ultrasonic clean		
	Air dry		

^a^Polymerization using calibrated LED light cure (Bluephase 20i, Ivoclar/Vivadent) at 1200 mW/cm^2^

### Thermocycling

All specimens were subjected to 3000 thermal cycles between 5 and 50 °C using a thermocycler machine (JULABO GmbH) with immersion time of 15 s in each bath.

### Color analysis

Color measurements of the specimens were performed before and after thermocycling by means of spectrophotometry (X-Rite). The CIE L*a*b* values of the specimens were measured according to the color change formula: *ΔE* = [(ΔL)2 + (Δa)2 + (Δb)2]1/2, where *ΔE* = color change; *Δ* = lightness difference (L*) such that the greater the L*, the higher the brightness of the sample; *Δa* = the a* axis difference such that the higher the a*, the redder the sample and the lower the a*, the greener the sample; and *Δb* = the b* axis difference such that the higher the b*, the yellower the sample and the lower the b*, the bluer the sample. *ΔL* = L*F − L*I; *Δa* = a*F − a*I; Δb = b*F − b*I, where L*I, a*I, and b*I represent the initial color measurement and L*F, a*F, and b*F represent the final color measurement.

### Statistical analysis

The Shapiro–Wilk test was used to test data for normal distribution. Parametric statistical tests were then performed. These were the paired t-test on individual L*, a*, and b* variables before and after thermocycling, two-way ANOVA on individual L*, a*, and b* variables for each test group, and one-way ANOVA to compare *ΔE* values for each study group. The confidence level was set as 95%. Data were expressed as mean ± standard deviation. Comparison was performed using SPSS version 20.0 for Windows. *p* < 0.05 was considered statistically significant.

## Results

For each study group, the mean and standard deviations of the color coordinates before and after thermocycling, and of the change in color (*ΔE*), are presented in Table 3. The overall mean change in color (*ΔE*) found in this study was 3.59 ± 1.60. All groups showed a normal distribution of data with the Shapiro–Wilk test (*p* > 0.05) except for the color change in the Zr/LC group (*p* = 0.031).

**Table 3:** Mean and standard deviations of color coordinates before and after thermocycling and of color change (*ΔE*) value

Group	N	Color coordinates before thermocycling mean ± SD			Color coordinates after thermocycling mean ± SD			*ΔE* mean ± SD
		L × I	a × I	b × I	L × F	a × F	b × F	
LiDi/LC	10	54.58 ± 1.44	− 1.16 ± 0.29	2.52 ± 1.58	58.01 ± 1.70	− 0.78 ± 0.36	4.21 ± 1.50	3.99 ± 1.83
LiDi/DC	10	56.78 ± 1.77	− 1.16 ± 0.22	3.73 ± 1.26	59.98 ± 2.15	− 0.65 ± 0.27	5.90 ± 1.68	3.67 ± 1.36
Zr/LC	10	64.80 ± 1.87	− 1.96 ± 0.17	3.74 ± 0.70	66.69 ± 2.86	− 1.43 ± 0.30	4.18 ± 0.98	3.22 ± 1.18
Zr/DC	10	63.96 ± 1.51	− 2 ± 0.22	2.91 ± 1.05	65.73 ± 20	− 1.61 ± 0.17	4.50 ± 1.18	3.49 ± 2.02
Total	40	60.03 ± 4.75	− 1.57 ± 0.47	3.22 ± 1.26	62.60 ± 4.30	− 1.12 ± 0.49	4.70 ± 1.49	3.59 ± 1.60

*LiDi* lithium disilicate, *LC* light-cure resin cement, *DC* dual-cure resin cement, *Zr* zirconia, *I* initial, *F* final. *SD* standard deviation

### Effect of thermocycling on color coordinates of luted ceramic material

**Fig. 1:**
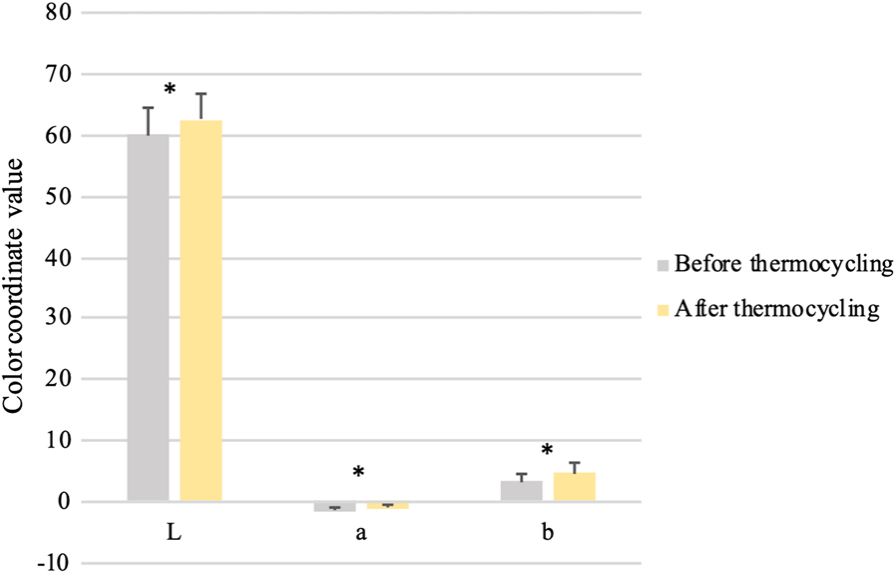
Means and standard deviations of color coordinates for ceramic specimens before and after thermocycling. *Statistically significant difference at *p* < 0.05

Thermocycling resulted in a significant change in the color coordinates of all tested specimens as determined by the paired t-test. Specimens became significantly brighter (*p* < 0.05), redder (*p* < 0.05), and more yellow (*p* < 0.05) after 3000 thermal cycles (Table 3; Fig. 1).

### Effect of ceramic/cement type on color coordinates of luted ceramic material

A two-way analysis of variance for the L* value showed a main effect for the ceramic type (*p* < 0.05) so that brightness was significantly higher for zirconia (66.21 ± 2.45) than for lithium disilicate (58.99 ± 2.14). The main effect of cement type was non-significant (*p* > 0.05). However, the interaction effect was significant (*p* < 0.05) indicating that ceramic type effect was greater with dual-cure cement than with light-cure cement for zirconia and greater with light-cure than dual-cure for lithium disilicate. Similarly, analysis for the a* value revealed a main effect for the ceramic type (*p* < 0.05) suggesting that redness was significantly higher for lithium disilicate (− 0.72 ± 0.06) than for zirconia (− 1.52 ± 0.06). However, the main effect of cement type and ceramic/cement interaction on the a* value were non-significant (*p* > 0.05). The two-way ANOVA test for the b* value showed a main effect for the cement type (*p* < 0.05). Therefore, yellowness was significantly higher for dual-cure resin cement (5.2 ± 0.3) than for light-cure resin cement (4.2 ± 0.3). The main effect of ceramic type or ceramic/cement interaction on the b* value was non-significant (*p* > 0.05).

### Comparison of color stability of luted ceramic material

**Fig. 2:**
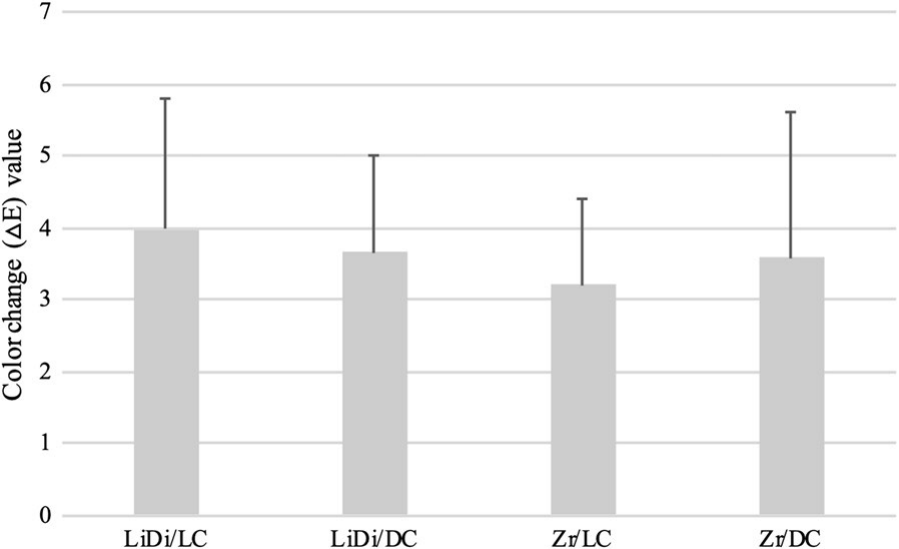
Means and standard deviations of color change (*ΔE*) value for ceramic specimens after thermocycling. *LiDi* IPS e.max CAD, *Zr* Katana Zirconia STML, *LC* light-cure resin cement, *DC* dual-cure resin cement. No statistically significant difference at *p* < 0.05

The one-way ANOVA test showed that there was no statistically significant difference in the mean color change (*ΔE*) values between all tested groups (*p* = 0.756) (Table 3; Fig. 2).

## Discussion

The current study utilized spectrophotometer to measure color coordinates of specimens. Color in dentistry can be evaluated using visual and/or instrumental methods. Although the visual method is subjective, it was the base for development of all color measuring instruments and it should complement their use. Instruments for color coordinate measurement include; spectrophotometer, colorimeters, and digital imaging systems. Spectrophotometer is considered one of the most accurate instruments for color measurement in dentistry. It measures the amount of light reflected from an object. This reflectance value is converted to shade tab equivalent. Colorimeter on the other hand is less accurate than spectrophotometer. It formulates color by filtering light in red, green, and blue areas of the visible spectrum. Additionally, digital cameras create a color image by acquiring red, green, and blue image information whereas software systems can compare shade in digital images with known reference shade [8].

The results of this study indicated that the color stabilities of the ceramic materials under the current investigation are comparable regardless of the type of luting cement used. Therefore, we failed to reject the null hypothesis. The current sample size was based on the reported mean and standard deviation of mean color change value among lithium disilicate, monolithic zirconia, and bilayer zirconia in Haralur et al*.* study [9] assuming alpha of 0.05 and a power of 80%.

Thermocycling resulted in a change in the color coordinates of all tested specimens. An increase in the L*, a*, and b* values indicates an increase in the brightness, redness, and yellowness of the specimen after cyclic aging, respectively. A change in the color of luted dental ceramics as a result of aging was reported in many previous studies [5–7, 10, 11]. In general, this can vary with aging conditions and can be attributed to an alteration in the color of the ceramic itself and/or the underlying cement. Ceramic material discoloration can be due to a loss of surface stain [12, 13], increased surface roughness, occurrence of surface cracks, change in ceramic translucency [13–16], and reduced ceramic thickness [5, 6]. On the other hand, resin cement discoloration can be manifested as a result of the degradation of unreacted polymers in the polymerization process. Therefore, the polymerization mode, time, cement shade, and composition all affect the cement color stability [7, 11]. Chaiyabutr et al. [17] reported that if a ceramic is less than 2 mm-thick, its optical color will be influenced by the underlying substrate color. In the current study, perhaps the high-translucency nature of the employed ceramics and the reduced thickness of 1 mm allowed for visualization of the color change of the underlying cement.

The overall mean color change value found in this study was 3.59 ± 1.6. The color difference is proposed to be perceptible when it can be detected by the human eye, and acceptable when it is tolerable [18]. Considering a threshold of perceptibility of *ΔE* = 1 and a threshold of acceptability of *ΔE* = 3.7 as concluded in a review by Khashayar et al. [19], the color change value in the current study is regarded perceptible but clinically acceptable for all specimens except for the lithium disilicate luted by the light-cure resin cement group (*ΔE* = 3.99 ± 1.83).

In our study, the color change values for specimens of IPS e.max CAD and Katana Zirconia STML were not significantly different (*p* = 0.756). Consistent with our findings, Subaci et al. [20] reported no significant difference in the color change values among three CAD-CAM monolithic ceramic materials. These were Vita Suprinity PC, IPS e.max CAD, and InCoris TZI C, all of the same thickness after 5000 thermocycles in a coffee solution. In contrast, several other studies demonstrated a significantly higher color change for zirconia than for lithium disilicate after artificial accelerated aging [9, 21]. In one investigation, in which lithium disilicate and zirconia underwent 3000 thermocycles between 5 °C and 55 °C in three discoloring solutions (coffee, green tea, and chlorohexidine gluconate), the ΔE values for zirconia were 5.60, 5.19, and 4.86 as compared to 1.78, 2.241, and 1.58 for lithium disilicate IPS Empress, respectively [9]. In another investigation, Kim et al. [21] reported that the color change for Katana monolithic zirconia was significantly higher than that of IPS e.max CAD following artificial aging in an autoclave at 134 °C under 0.2 MPa for 0, 1, 3, 5, or 10 h. Inconsistencies among findings in the literature on the color stability of ceramic materials can be attributed to variations in the specimen preparation and aging methods as well as to the diversity in material composition and optical properties. In high-translucency zirconia, such as the type used in this study and in Subaci’s study [20], the presence of at least 5.5 mol% of yttria increases the cubic phase content, which is responsible for the improved translucency and the lack of hydrothermal degradation under in vitro aging conditions when no load is applied [22]. This absence of low-grade thermal degradation, and its consequences of surface roughness and cracks, is speculated to improve the color stability of the highly translucent zirconia as compared to the low-translucency type.

In the present investigation, the brightness was significantly higher for zirconia samples than for lithium disilicate samples, due to ceramic type effect. The increased brightness of zirconia may be related to its higher refractive index, inducing more scattering of the light passing through, and to its lower translucency than lithium disilicate [23–25].

Additionally, the present study demonstrated that samples luted with dual-cure resin cement were significantly more yellow than samples luted using light-cure resin cement. The main contributor to this difference was the cement type. Consistent with our finding, several previous studies reported that dual-cure resin cement is more yellow than light-cure resin cement upon polymerization [11]. This was probably attributed to the degree of oxidation of the unreacted amine accelerators, causing their discoloration. The resultant discoloration was found to be greater in dual-cure than in light-cure resin cements [11, 26]. However, in the current study, both NX3 Nexus light-cure and dual-cure resin cements utilize an amine-free initiator system for better color stability. Nevertheless, the significant effect of the cement type on the b* value may indicate that these two cements degrade differently.

The results of the present study could have been enhanced by performing measurements of the substrate color coordinate and specimens transparency parameter, in addition to increasing the current sample size and number of thermal cycles. Further studies should be conducted on the optical and mechanical properties of the high-translucency ceramic materials to delineate their recommended clinical situations.

## Conclusions

Within the limitations of this in vitro study, the following conclusions could be drawn:

- Thermocycling resulted in a significant change in the color coordinates of all tested specimens.
- The overall mean color change value found in this study was considered within the clinically acceptable level.
- At 1 mm restoration thickness, the color stabilities of high translucency monolithic lithium disilicate and high-translucency zirconia ceramics were not significantly different, regardless of the cement type used.

## Data Availability

The datasets used and/or analyzed during the current study are available from the corresponding author on reasonable request*.*
